# The serodiagnositic value of *Chlamydia trachomatis* antigens in antibody detection using luciferase immunosorbent assay

**DOI:** 10.3389/fpubh.2024.1333559

**Published:** 2024-02-27

**Authors:** Yulian Pang, Jingwei Shui, Changchang Li, Yongzhi Li, Hongliang Chen, Shixing Tang

**Affiliations:** ^1^Department of Epidemiology, School of Public Health, Southern Medical University, Guangzhou, Guangdong, China; ^2^Department of Emergency, The Second Affiliated Hospital of Guangzhou University of Chinese Medicine, Guangzhou, Guangdong, China; ^3^Dermatology Hospital of Southern Medical University, Guangzhou, China; ^4^Chenzhou No.1 People's Hospital, Chenzhou, China

**Keywords:** *Chlamydia trachomatis*, luciferase immunosorbent assay (LISA), plasmid-encoded protein 3 (Pgp3), translocated membrane-associated effector A (TmeA), inclusion membrane protein for actin assembly (InaC), heat shock protein 60 (HSP60)

## Abstract

**Introduction:**

Among the different antigens used in the detection of anti-*Chlamydia trachomatis* antibodies, significant differences in sensitivity and specificity have been observed. Further evaluation of *C. trachomatis* antigens in antibody detection is urgently needed for the development and application of *C. trachomatis* serologic assays.

**Methods:**

*Chlamydia trachomatis* antigens Pgp3, TmeA, InaC, and HSP60 were selected and used in luciferase immunosorbent assay (LISA). The detection results obtained from well-defined *C. trachomatis* positive and negative samples were compared with the commercial *C. trachomatis* ELISA (Mikrogen) for performance evaluation.

**Results:**

Pgp3, TmeA, InaC, and HSP60-based LISA showed sensitivity of 92.8, 88.8, 90.4, and 94.4%, and specificity of 99.2, 99.2, 99.2, and 92%, respectively. ROC analysis indicated that Pgp3-based LISA showed similar performance to Mikrogen ELISA (AUC 0.986 vs. 0.993, p = 0.207). Furthermore, four *C. trachomatis* antigens achieved strong diagnostic efficiency, i.e., positive likelihood ratios [+LR] ≥ 10 in *C. trachomatis*-infected women and negative likelihood ratios [−LR] ≤ 0.1 in *C. trachomatis* negative low exposure risk children, but only Pgp3 and TmeA showed strong diagnostic value in general adults. In addition, Pgp3, TmeA, and InaC, but not HSP60, achieved high performance, i.e., both positive predictive value (PPV) and negative predictive value (NPV) ≥ 90.9%, and showed no significant cross-reactivity with anti-*Chlamydiapneumoniae*.

**Conclusion:**

Three *C. trachomatis* species-specific antigens Pgp3, TmeA, and InaC show superior performance in the detection of anti-*C. trachomatis* antibody, indicating the potential to be used in developing *C. trachomatis* serologic tests.

## 1 Introduction

*Chlamydia trachomatis*, an obligate intracellular bacterium, widely spreads and can cause both urogenital and ocular infections (1). *Chlamydia trachomatis* can be classified into 19 genotypes according to the diversity of its OmpA gene sequences (2, 3). Genotypes D-K mainly cause genital infection, the most common bacterial sexually transmitted infection, with an annual 131 million new cases worldwide (4). Unfortunately, most genital chlamydial infections are asymptomatic and undetected, resulting in continued transmission and delayed or no treatment (5). Untreated *C. trachomatis* infection is able to cause more serious complications, especially pelvic inflammatory disease, ectopic pregnancy, and tubal factor infertility in women (1, 6, 7). Additionally, *C. trachomatis* infection may enhance human immunodeficiency virus (HIV) infection and help develop cervical cancer (8–10). However, screening and timely treatment of *C. trachomatis* infection are still an unmet goal. Currently, the diagnosis of *C. trachomatis* infection primarily relies on nucleic acid amplification tests (NAATs), which are resource and labor intensive, and difficult for routine diagnosis in resource-limiting settings (11). Furthermore, NAAT results mean current and active infection of *C. trachomatis* (12, 13), and cannot determine the prevalence of *C. trachomatis* exposure and disease burden unless serological assays are used in epidemiological or retrospective analyses (14, 15). *Chlamydia trachomatis* serology is also useful in understanding the natural history of *C. trachomatis* and providing support for the development and evaluation of *C. trachomatis* vaccines (1, 16).

However, a major issue for *C. trachomatis* serological detection is the cross-reactivity between *C. trachomatis* and other *Chlamydia* spp., especially *Chlamydia pneumoniae*. Microimmunofluorescence (MIF), which is the “gold standard” for serodiagnosis of chlamydial infection, is affected by the cross-reactivity of antibodies against *C. trachomatis* elementary body (EB) antigens with those against other *Chlamydia* species (17–19). The most commonly used *C. trachomatis* antigens, such as EBs, lipopolysaccharide (LPS), and major outer membrane protein (MOMP), also show high cross-reactivity in the simple enzyme-linked immunosorbent assay (ELISA) format due to genus-specific B cell epitopes (20–22). Although recombinant antigens of *C. trachomatis*-specific peptides can reduce cross-reactivity, the corresponding assays are usually not sensitive enough with a sensitivity from 45.7% (IgG pELISA plus medac assay) to 82.9% (Pgp3 double-antigen) in women, and from 40.0% (SeroCT) to 54.4% (Pgp3 double-antigen) in men when compared with NAAT (23–27). Although combined use of peptides of *C. trachomatis*-specific B cell epitopes could increase the sensitivity to 91.8%, testing IgG1 and IgG3 antibodies against 11 peptides is too complicated to apply in population screening (28). In general, the immunogenicity of *C. trachomatis*-specific peptides is much less than that of intact *C. trachomatis* antigens. Recently, several *C. trachomatis* species-specific antigens have been used in serological assays to improve their specificity. Among them, plasmid-encoded protein 3 (Pgp3) is a promising *C. trachomatis* species-specific and immunodominant antigen since it is highly conserved across *C. trachomatis* strains and rarely identified in *C. pneumoniae* (29–31). Pgp3-based ELISA methods show a sensitivity of 53.0–80.9% and a specificity of 80.0–98.0% (20, 23, 27, 32–39). In addition, translocated membrane-associated effector A (TmeA) encoded by CT694 gene and inclusion membrane protein for actin assembly (InaC) encoded by CT813 gene are species-specific antigens of *C. trachomatis* since there are no known homologous proteins in *C. pneumoniae* (40, 41). TmeA is an effector protein of the type III secretion system that facilitates *C. trachomatis* invasion (42–45). Antibody assays using TmeA as the antigen show a sensitivity of 30.8–91.0% and a specificity of 69.4–98.0% (41, 44). InaC is expressed on the membrane of *C. trachomatis* inclusion bodies and can polymerize the cytoskeleton of host cells to regulate *C. trachomatis* reproduction (46, 47). InaC-based ELISA also shows a sensitivity of 60.0% and a specificity of 100.0% (48). At present, an unresolved problem is the significant difference in detection sensitivity and specificity between the assays based on different *C. trachomatis* species-specific antigens due to multiple factors including lack of standardized evaluation and well-defined positive and negative controls, differences in assay format and antigens (1, 39).

We have previously reported a high-throughput luciferase immunosorbent assay (LISA) for the qualitative and semi-quantitative detection of antibodies, which is more convenient, straightforward and highly sensitive (49). In the current study, we introduced *C. trachomatis* species-specific and immunodominant antigens including Pgp3, TmeA, and InaC into our “in-house” LISA to evaluate its serodiagnositic value. In addition, heat shock protein 60 (HSP60), another antigen commonly used in *C. trachomatis* immunoassay, was selected to provide complementary information to better characterize the assays since it is one of the most conserved proteins in evolution. Previous studies indicated that anti-HSP60 antibody is associated with tubal factor infertility (TFI) (50, 51) although it has been found to have cross-reactivity with *C. pneumoniae* (32).

## 2 Materials and methods

### 2.1 Serum samples

A total of 450 serum samples collected from Chenzhou No.1 People’s Hospital, Guangzhou Dermatology Hospital and The Third Affiliated Hospital of Southern Medical University were used in this study. Group 1 contains 125 serum samples from *C. trachomatis* infected women who are *C. trachomatis* positive by NAAT, and 125 plasma specimens collected from healthy children aged 1–6 years old with low exposure risk to *C. trachomatis* and anti*-C. trachomatis* IgG negative. Samples of group 2 were collected from 200 general adults whose *C. trachomatis* infection was unknown. The characteristics of these subjects were shown in Supplementary Table S1. All the samples were stored at −80°C until they were processed. The study followed the ethical recommendations of the Declaration of Helsinki and obtained the informed consent of the participants and the parents or guardians of the children. Ethics Committee of Dermatology Hospital of Southern Medical University approved this study (GDDHLS-20181207).

Nucleic acid amplification test for screening *C. trachomatis* nucleic acid-positive women was performed by using cobas® 4800 CT/NG Amplification/Detection Kit (Roche Diagnostics, United States) in Guangzhou Dermatology Hospital or by using home-made reagents in Chenzhou No.1 People’s Hospital. The homemade NAAT assay used specific primers based on the highly conserved cryptic plasmid Pgp2 gene of *C. trachomatis* and included forward primer of 5′-TTCCCCTTGTAATTCGTTGC-3′ and reverse primer of 5′-TAGTAACTGCCACTTCATCA-3′. After DNA extraction from the patients’ cervical swabs by using QIAamp DNA Mini Kit (Qiagen, Germany), target nucleic acid was amplified by PCR. Briefly, 12.5 μL Taq Plus Master Mix (CoWin Biotechnology Co., Ltd., JiangSu, China), 0.5 μL of forward and reverse primers (10 μM), 9.5 μL of nuclease-free water, and 2 μL of target template were mixed. The reaction was run at 95°C for 5 min followed by 35 cycles of 95°C for 50 s, 55°C for 45 s, and 72°C for 45 s.

### 2.2 *Chlamydia trachomatis* antigen-based luciferase immunosorbent assay

Four *C. trachomatis* antigens Pgp3, TmeA, InaC, and HSP60 (Supplementary Table S2) were amplified and cloned into pNLF1-N luciferase expression vector (Promega, Madison, WI, United States) downstream of the Nluc luciferase gene as previously described (49). All materials and reactive used in this study were consistent with those of Wang. All recombinant plasmids were confirmed by DNA sequencing and transfected into HEK-293 T cells (ATCC CRL-3216). The expressed fusion proteins of Nluc-*C. trachomatis* in cell lysates were harvested for further confirmation using anti-luciferase antibody, and then stored at −80°C until use. Anti-*C. trachomatis* antibodies in sera were detected in LISA in which they were first captured by protein G-coated microtiter plate and detected by Nluc-*C. trachomatis* antigen lysate in the presence of luciferase substrate as described previously (49). Each sample was tested in triplicate and the relative light units (RLU) were calculated by dividing each sample’s average luciferase light units with the average LU of the control wells. The level of anti-*C. trachomatis* antibody was expressed as Log_2_RLU. The cut-off value of *C. trachomatis* LISA was determined according to the maximum value of Youden’s index obtained through the receiver operating characteristic (ROC) curve.

### 2.3 Commercial *Chlamydia trachomatis*/*Chlamydia pneumoniae* enzyme linked immunosorbent assay

A commercial recomWell *C. trachomatis* IgG ELISA kit that can detect antibodies against MOMP, translocated actin-recruiting phosphoprotein (TARP) and chlamydial protease-like activity factor (CPAF) was used to detect anti-*C. trachomatis* IgG in the sera. RecomWell *Chlamydia pneumoniae* IgG ELISA kit that detects antibodies against the outer membrane complex of elementary bodies and reticulate bodies of *Chlamydia pneumoniae* (COMC) was used for evaluating the specificity of *C. trachomatis* LISA. The ELISA kits were purchased from Mikrogen (Mikrogen GmbH, Neuried, Germany), and the cut-off values were determined according to the manufacturers’ instructions.

### 2.4 Determination of likelihood ratio and predictive value of *Chlamydia trachomatis* LISA

The positive likelihood ratio (+LR) and negative likelihood ratio (−LR) were calculated as sensitivity/(1 − specificity) and (1 − sensitivity)/specificity, respectively according to the sensitivity and specificity data of ROC curves. +LR ≥ 10 and −LR ≤ 0.1 indicate strong evidence to rule in or rule out the diagnosis, respectively (28). For different prevalence, positive and negative predictive values (PPV and NPV) were calculated as PPV = [sensitivity × prevalence] ÷ [(sensitivity × prevalence) + (1 − specificity) × (1 − prevalence)] while NPV = [specificity × (1 − prevalence)] ÷ [(1 − sensitivity) × prevalence + specificity × (1 − prevalence)] (52).

### 2.5 Statistical analysis

All data were analyzed using IBM SPSS 27.0 (SPSS Inc., Chicago, United States) and GraphPad Prism 8.0.2 (GraphPad Software, California, United States). Sensitivity, specificity and kappa coefficient were calculated by using two-by-two tables, and McNemar test were used to compare the difference of sensitivity and specificity between assays. The kappa coefficient was used to assess the agreement between different assays (53). Receiver operating characteristic (ROC) analysis was performed to determine the optimized cut-off values and calculate the area under ROC curves (AUCs). Delong test was used to compare ROC curves while Student’s *t*-tests were used to compare the average sensitivity between different assays. False positive rates were compared by Chi-square tests. *p* values ≤0.05 were considered statistically significant.

## 3 Results

### 3.1 Detection of anti-*Chlamydia trachomatis* IgG antibodies by ELISA and LISA

For the 125 serum samples from women with active *C. trachomatis* infection, 93.6% (117/125) were anti-*C. trachomatis* IgG positive by Mikrogen ELISA while none of the 125 children sera were positive. Then, ROC analysis was conducted to determine the optimal cut-off value for each *C. trachomatis* antigen-based LISA. Based on the cut-off values we selected, the detection sensitivity was 92.8, 88.8, 90.4, and 94.4% for Pgp3, TmeA, InaC, and HSP60-based LISA, respectively, and the corresponding specificities were 99.2, 99.2, 99.2, and 92.0%, respectively (Table 1). Pgp3, TmeA, and InaC-based LISA showed similar sensitivity and specificity to Mikrogen-CT ELISA whereas HSP60-based LISA showed significantly lower specificity (92.0%, *p* = 0.002). Pgp3-based LISA showed the best agreement with Mikrogen-CT ELISA (kappa = 0.920) among the four *C. trachomatis* antigens analyzed.

**Table 1:** Performance of different *Chlamydia trachomatis* antigen-based antibody detection.^a^

*C. trachomatis* antigen	No. of samples tested				Sensitivity ^c^ (%)	*p* value ^d^	Specificity (%)	*p* value	Kappa
	True positive ^b^	False positive	True negative	False negative					
Mikrogen	117	0	125	8	93.6	Ref ^e^	100.0	Ref	0.936
Pgp3	116	1	124	9	92.8	>0.999	99.2	>0.999	0.920
TmeA	111	1	124	14	88.8	0.264	99.2	>0.999	0.880
InaC	113	1	124	12	90.4	0.485	99.2	>0.999	0.896
HSP60	118	10	115	7	94.4	>0.999	92.0	0.002	0.864

^a^Sera from 125 women with *Chlamydia trachomatis* NAAT positive and 125 children with anti-*C. trachomatis* antibody negative were used as positive and negative controls to evaluate the performance of different *C. trachomatis* antigen-based antibody detection.

^b^True positive of anti-*Chlamydia trachomatis* was defined as positive results detected by Mikrogen ELISA or LISA in the positive control while true negative was defined as negative results detected by Mikrogen ELISA or LISA in the negative control. False positive was defined as positive results detected by Mikrogen ELISA or LISA in the negative control while false negative was defined as negative results detected by Mikrogen ELISA or LISA in the positive control.

^c^For Mikrogen ELISA, the cut-off value was defined by manufacturer while for home-made LISA, the cut-off value was determined by ROC analysis.

^d^Two-sided McNemar’s test.

^e^Mikrogen-CT ELISA was set as reference.

Of note, 5.6% (7/125) of the sera from actively *C. trachomatis*-infected women were anti-*C. trachomatis* antibody negative by both CT ELISA and LISA (Supplementary Figure S1), probably due to window period, i.e., NAAT positive but antibody negative during very early acute *C. trachomatis* infection. In addition, there were only one child’s serum positive by Pgp3-LISA, one child’s serum positive by TmeA-LISA, one child’s serum positive by InaC-LISA, and 10 children’s sera positive by HSP60-LISA, respectively. However, no children’s sera were positive for any two *C. trachomatis* antigen-LISAs simultaneously, suggesting a false positivity.

### 3.2 Performance of *Chlamydia trachomatis* antigen-based LISA

**Figure 1:**
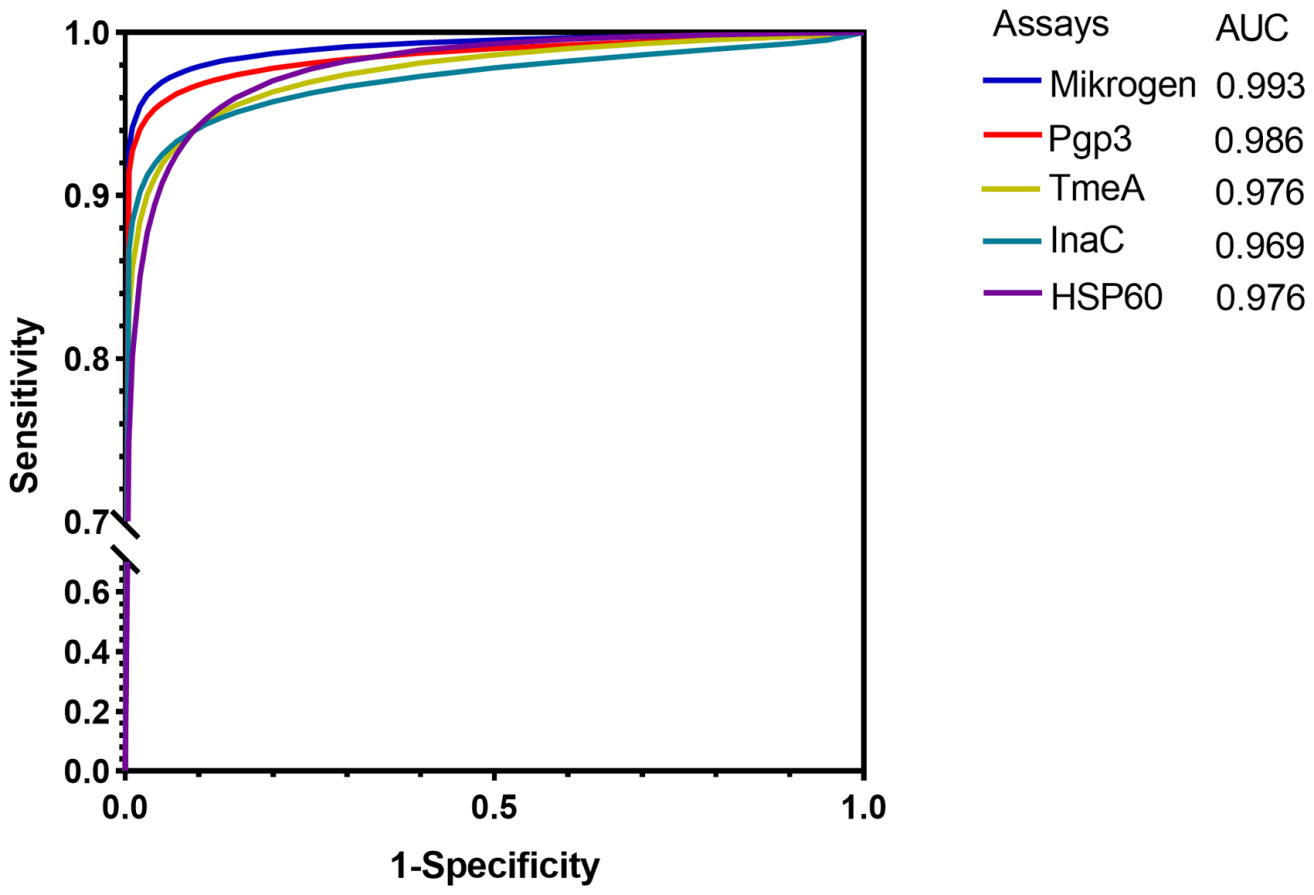
ROC curves of different *Chlamydia trachomatis* antigen-based LISA and commercial ELISA in detecting anti-*C. trachomatis* antibody. 125 *C. trachomatis* NAAT-positive and 125 *C. trachomatis* negative from low exposure risk children sera were used. The average Log2RLU signals of LISAs and the OD values of commercial ELISA were used as predictor variables. The solid color lines indicate the maximum likelihood-fitted ROC curves.

When the specificity was set as 90, 95, and 98%, Pgp3-based LISA achieved the highest sensitivity with an average sensitivity of 95.5%, followed by InaC (91.6%), TmeA (92.3%), and HSP60 (90.0%), respectively (Table 2). There was no significant difference for the detection sensitivity between Mikrogen ELISA and Pgp3 or InaC-based LISA (for Pgp3, 96.8 vs. 95.5%, *p* = 0.585; for InaC, 92.3 vs. 95.5%, *p* = 0.072). However, TmeA and HSP60-based LISA showed significantly lower average sensitivity (for TmeA, 91.6%, *p* = 0.041; for HSP60, 90.0%, *p* = 0.013). ROC-AUC analysis, a cutoff-independent global assay performance measurement, on the other hand, found no statistically significant difference in performance between Pgp3-based LISA and Mikrogen ELISA (AUC 0.986 vs. 0.993, *p* = 0.207, Table 2; Figure 1).

**Table 2:** Prediction of seroreactivities of *Chlamydia trachomatis* antigen-based antibody detection.^a^

*C. trachomatis* antigen	Sensitivity (%) based on different specificity^b^				*p* value ^c^	AUC ± SE ^e^	*p* value^f^
	Specificity = 90%	Specificity = 95%	Specificity = 98%	Average			
Mikrogen	97.9	97.0	95.5	96.8	Ref ^d^	0.993 ± 0.0031	Ref
Pgp3	96.8	95.7	94.1	95.5	0.585	0.986 ± 0.0056	0.207
TmeA	94.3	91.9	88.5	91.6	0.041	0.976 ± 0.0075	0.006
InaC	94.2	92.5	90.2	92.3	0.072	0.969 ± 0.0107	0.005
HSP60	94.3	90.8	85.1	90.0	0.013	0.976 ± 0.0095	0.065

^a^125 *Chlamydia trachomatis* NAAT positive and 125 *C. trachomatis* negative sera from low exposure risk children were used to evaluate the performance of different *C. trachomatis* antigen-based antibody detection.

^b^The sensitivities of each assays at different specificity cutoff values were calculated by ROC analysis.

^c^Comparisons of the average sensitivity was carried out by Student’s *t*-test.

^d^Mikrogen-CT ELISA was set as reference.

^e^Area under ROC curve, an AUC value of 0.50 indicates that discrimination of positive from negative data equals random categorization (no discrimination), and an AUC value of 1.0 indicates that prediction is perfect (100% accurate discrimination). SE, standard error.

^f^Comparisons of AUC between assays was carried out by Delong test.

### 3.3 Diagnostic utility of *Chlamydia trachomatis* antigen-based LISA

**Figure 2:**
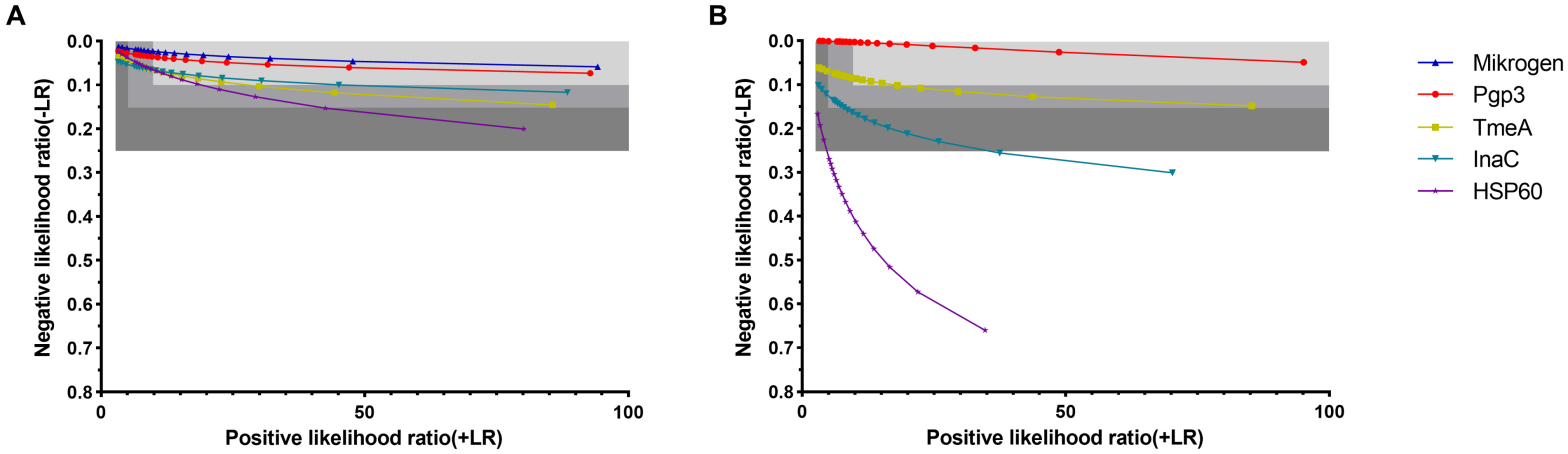
Diagnostic utility of anti-*Chlamydia trachomatis* antibody assays by likelihood ratios in control sera **(A)** and general adults **(B)**. Sensitivities were calculated at specificities ranging from 70% (left) to 99% (right). Using sensitivity and specificity data of ROC curves which *C. trachomatis* exposure status was set as the gold standard for control sera and the determination of Mikrogen ELISA was set as the gold standard for general adults, positive-likelihood ratios (+LR) and negative-likelihood ratios (−LR) were calculated. The three-gray shaded areas indicate the zones of strong (+LR ≥ 10, −LR ≤ 0.1), moderate (+LR ≥ 5, −LR ≤ 0.15), and poor (+LR ≥ 2.5, −LR ≤ 0.25) performance of the assays, corresponding to the strong, moderate and poor diagnostic efficiencies.

When detecting anti-*C. trachomatis* antibody in active *C. trachomatis* infected women and anti-*C. trachomatis* negative low exposure risk children, at the specificity level of 91–99%, the corresponding sensitivity was 97.8–94.2% for Mikrogen ELISA and 96.6–92.8% for Pgp3-based LISA while the positive-likelihood ratios were ≥ 10 and the negative-likelihood ratios were ≤ 0.1 (Figure 2A; Supplementary Table S3). Similar results were obtained for TmeA, InaC, and HSP60-based LISA with the corresponding sensitivity of 93.9–91.1, 93.9–90.2, and 93.8–90.8%, respectively (Figure 2A; Supplementary Table S3), indicating strong diagnostic efficiency for both Mikrogen ELISA and *C. trachomatis* antigen-based LISA.

When detecting anti-*C. trachomatis* antibody in general adults, Pgp3 and TmeA-based LISA showed strong diagnostic efficiency with specificity of 91–99 and 91–94%, respectively whereas InaC-based LISA showed only moderate diagnostic efficiency with specificity of 85–88% (Figure 2B; Supplementary Table S3). However, HSP60-based LISA showed poor diagnostic efficiency with +LR ≥ 2.5 and -LR ≤ 0.25, indicating a significantly lower diagnostic power in general adults.

### 3.4 Predictive value of *Chlamydia trachomatis* antigen-based LISA depends on the positive rate or prevalence of anti-*Chlamydia trachomatis*

**Figure 3:**
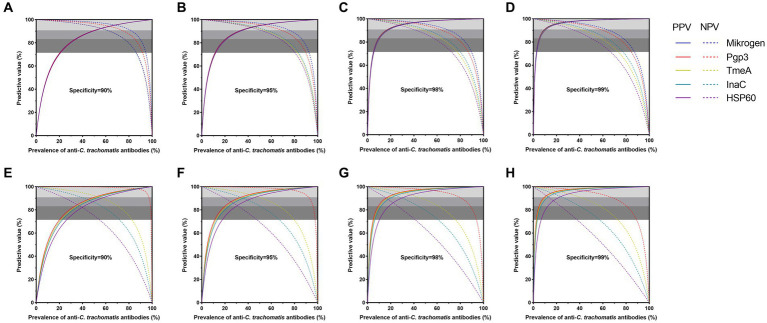
Diagnostic suitability of anti-*Chlamydia trachomatis* antibody assays by predictive values in control sera **(A–D)** and general adults **(E–F)**. Positive and negative predictive values are dependent on the prevalence of populations of anti-*C. trachomatis* antibodies. Four specificity cut-off values and resultant sensitivities in ROC curve analysis were selected for panels (**A,E**; 90% specificity), (**B,F**; 95%), (**C,G**; 98%), and (**D,H**; 99%). The three-gray shaded areas indicate the zones of high (PPV and NPV ≥ 90.9%), moderate (PPV and NPV ≥ 83.3%), and poor (PPV and NPV ≥ 71.4%) diagnostic efficiency of assays.

Next, we adapted the predictive value model to compare and evaluate the diagnostic suitability of *C. trachomatis* antigen-based LISA at different levels of anti-*C. trachomatis* positivity or prevalence. All four *C. trachomatis* antigen-based LISAs and Mikrogen ELISA achieved high performance with both PPV and NPV ≥ 90.9% at the specificity level of 90–99%, in women with active *C. trachomatis* infection and anti-*C. trachomatis* negative low exposure risk children (Figures 3A–D). Furthermore, when the assay specificity increased, the optimal prevalence range changed. For example, the prevalence of anti-*C. trachomatis* that could be achieved by Mikrogen ELISA ranged from 51 to 81% at the specificity level of 90%, and from 10 to 63% at 99% specificity, indicating 23% increase of the optimal prevalence range when the specificity increased from 90 to 99% (Supplementary Table S4). At the specificity of 99%, the prevalence range of anti-*C. trachomatis* for the assays tested ranked as Mikrogen ELISA (10–63%) > Pgp3-based LISA (10–57%) > InaC-based LISA (11–46%) > TmeA-based LISA (11–40%) > HSP60-based LISA (12–33%) (Figure 3; Supplementary Table S4).

Of note, further predictive value analysis of anti-*C. trachomatis* antibody detection assays was conducted in general adults to explore its feasibility in general population with relatively low prevalence of *C. trachomatis* infection. High performance was achieved for Pgp3-based LISA with a specificity of 90–99% while the specificity for TmeA-based LISA was 90–99% and InaC-based LISA was 98–99% at different positive rates or prevalence levels of anti-*C. trachomatis* antibody. However, HSP60-based LISA only performed moderately well (PPV and NPV ≥ 83.3%) at the specificity of 98–99% (Figures 3E–H; Supplementary Table S4). At the specificity level of 99%, Pgp3-based LISA showed the largest prevalence range of 10–67%, followed by TmeA-based LISA (11–40%) and InaC-based LISA (13–24%, Figures 3E–H; Supplementary Table S4).

### 3.5 Cross-reactivity of *Chlamydia trachomatis* antigen-based LISA with anti-*Chlamydia pneumoniae* ELISA

Of the 162 anti-*C. trachomatis* negative sera from general adults, 40 (24.7%) were positive for anti-*C. pneumoniae* antibody (Table 3). HSP60-based LISA showed high false positivity of anti-*C. trachomatis* antibody when testing anti-*C. pneumoniae* positive samples (*p* = 0.028; Table 3). No cross-reactivity with anti-*C. pneumoniae* positive samples were observed for Pgp3, TmeA, and InaC-based LISA (*p* > 0.4; Table 3), demonstrating the high specificity of the three *C. trachomatis* species-specific antigens.

**Table 3:** Antibody cross-reactivity between *Chlamydia trachomatis* and *Chlamydia pneumoniae* assay.

*C. trachomatis* antigen	Anti-*C. trachomatis* positive (*n*, %) ^a^		*p* value^b^
	Anti-CP IgG positive	Anti-CP IgG negative	
	*N* = 40	*N* = 122	
Pgp3	1 (2.5)	6 (4.9)	0.838
TmeA	0	0	0.439
InaC	3 (7.5)	4 (3.3)	0.489
HSP60	18 (45.0)	31 (25.4)	0.028

^a^40 anti-*C. pneumonia* positive and 122 anti-*C. pneumoniae* negative samples, but anti-*C. trachomatis* antibodies negative determined by Mikrogen-CT ELISA, were used for the analysis.

^b^*p* values were calculated using Chi-square tests.

## 4 Discussion

In the present study, we comprehensively evaluated the performance of *C. trachomatis* antigen-based LISAs in sera from current *C. trachomatis*-infected women, anti-*C. trachomatis* negative children with low-risk exposure to *C. trachomatis*, and general adults with unknown *C. trachomatis* infection status to analyze the diagnostic value of different *C. trachomatis* antigens including Pgp3, TmeA, InaC, and HSP60 in *C. trachomatis* serological testing. Our results demonstrated the great performance of all four *C. trachomatis* antigens in testing both *C. trachomatis* positive and negative sera while only Pgp3 and TmeA-based LISA performed well in detecting anti-*C. trachomatis* antibodies in general adults. Our data confirmed that *C. trachomatis* species specific antigen Pgp3 exhibited excellent diagnostic value in different populations according to its high sensitivity, specificity, PPV and NPV as well as the wide range of anti-*C. trachomatis* prevalence. Pgp3 has widely been used as a detecting antigen in serological assays including ELISA, multiplex bead array, and point-of-care testing, with sensitivity of 44.2–92.0% and specificity of 80.0–98.0% (20, 23, 27, 32–34, 39, 54, 55). When evaluating in a large population, a sensitivity of 82.9% and a specificity of 97.8% were reported for a double-antigen Pgp3 ELISA in 158 *C. trachomatis*-positive women and 494 *C. trachomatis*-negative pediatric sera (23). In our study, the Pgp3-based LISA showed a sensitivity of 92.8% and specificity of 99.2% in 125 *C. trachomatis* NAAT-positive women and 125 *C. trachomatis* negative children, indicating the potential and advantage of Pgp3 as a *C. trachomatis* species-specific antigen and immunodominant protein in the development of antibody detection assays including LISA.

Evaluation of anti-*C. trachomatis* serological assays relies on the selection of serum samples. In general, *C. trachomatis* NAAT-positive samples are commonly chosen as positive control in which more than 90% of NAAT-positive samples were positive for *C. trachomatis*-specific IgG antibodies (27, 28, 32). In the current research, we included 125 *C. trachomatis* NAAT-positive samples as positive controls and 125 anti-*C. trachomatis* antibody negative sera in children aged 1–6 years old as negative controls since children are considered at lower risk of exposure to *C. trachomatis* (27). In the current research, we included 125 *C. trachomatis* NAAT-positive samples as positive controls and 125 anti-*C. trachomatis* antibody negative sera in children aged 1–6 years old as negative controls. We used the commercial Mikrogen ELISA *C. trachomatis* kit instead of NAAT to screen negative children, since anti-*C. trachomatis* antibody can persist for many years when NAAT becomes negative (23) while anti-*C. trachomatis* antibody may be negative at the window period when NAAT becomes positive, and our study is mainly focused on the antibody detection of *C. trachomatis* rather than to determine the infection status of *C. trachomatis*. We found that 93.6% of NAAT positive sera determined positive for *C. trachomatis* IgG antibodies, which was consistent with previous study (56). Lack of anti-*C. trachomatis* specific antibody response has previously been reported in 5.5–10.2% of *C. trachomatis*-infected subjects (57–60) and can be explained by several possible reasons including early acute infection, delayed seroconversion, reduced infection duration, and antigenic burden caused by early treatment, or transient infection due to inadequate infection dose of *C. trachomatis*, or undiagnosed immunosuppressive diseases (58). Of note, of the 125 children sera, 0.8% were seropositive for by Pgp3, TmeA, or InaC-based LISA while 8.0% were positive by HSP60-based LISA. Wills et al. (27) also reported the *C. trachomatis* seropositive rate of 3.3% in pediatric sera by MIF, which may be due to the vertical transmission of *C. trachomatis* infection (61) or *C. trachomatis* eye disease (62). However, in our study, the positive results observed in children were more likely to be false positive as none was positive for any two *C. trachomatis* antigens simultaneously.

By using these well-defined samples, we found that TmeA-based LISA showed a sensitivity of 88.8% and a specificity of 99.2%, which is similar to earlier studies (41, 46). Wang et al. found that anti-TmeA antibody could be detected in approximately 80.8% (80/99) of *C. trachomatis* NAAT-positive sera. A TmeA-based multiplex bead assay showed a sensitivity of 90.9% and a specificity of 98.4% by using samples collected from 11 *C. trachomatis* PCR-positive children in Tanzania and 122 children from non-endemic areas in the United States. In addition, we found a sensitivity of 90.4% in our InaC-based LISA in this study, which is significantly higher than the sensitivity of 79% by the InaC-based ELISA in the study of Wang et al. (46), and 60% in the study of Gao et al. (48). The difference may be due to the relatively higher sensitivity of LISA than ELISA as observed in the previous study (49). The relative lower sensitivity and specificity of HSP60-based assays have also been reported (32).

Besides sensitivity, specificity, and AUC values, we also adopted likelihood ratio, predictive values, and range of antibody prevalence to evaluate the performance of *C. trachomatis* antigen-based LISAs. Likelihood ratio is a comprehensive index of both sensitivity and specificity whereas the predictive value can reflect the benefit gained from the actual application of diagnostic tests (63). The reliable and useful serological assays should be able to maintain a high predictive value across a wide range of antibody prevalence as well as a high positive likelihood ratio and a low negative likelihood ratio (28). Based on the likelihood ratios and predictive values we observed, the four *C. trachomatis*-antigen LISAs and the commercial Mikrogen ELISA showed moderate to strong diagnostic efficiency in detecting current *C. trachomatis*-infected women and children with low exposure risk to *C. trachomatis*. However, when detecting general adults, HSP60-based LISA showed poor diagnostic efficiency probably due to cross-reactivity with *C. pneumoniae*. Pgp3-based LISA, in contrast, maintained strong diagnostic efficiency even in general adults, suggesting its potential as a robust assay (28). TmeA-based LISA also achieved strong diagnostic efficiency with 91–94% specificity and 92.2–90.9% sensitivity while the antibody prevalence ranged from 36 to 54%, which is higher than the prevalence range of 20–30% in general population reported previously (23, 64–66), which suggests its feasibility in screening population with high prevalence of *C. trachomatis* infection (67).

The cross-reactivity between *C. trachomatis* and *C. pneumoniae* remains a basic problem in serological assays. However, except for HSP60, our results suggest that *C. pneumoniae* exposure had limited influence on the LISAs based on Pgp3, TmeA, and InaC, likely because these are *C. trachomatis*-specific antigens and have no equivalent orthologues in *C. pneumoniae*, which were consistent with previous studies (27, 40, 46). In contrast, HSP60, a highly-conserved protein that shares 89–95% identity with *C. pneumoniae* (28, 68, 69), showed strong cross-reactivity with *C. pneumoniae*. However, the cross-reactivity with *C. pneumoniae* cannot explain the high false positive rate of 25% observed in the *C. pneumoniae* negative group, suggesting that there may be other unrevealed factors, such as the cross-reactivity with human HSP60, which shares 48% identity with *C. trachomatis* (70, 71).

There are some limitations in our study. First, there is no recognized gold standard method for antibody detection of *C. trachomatis*. In our study, we only used one commercially available ELISA kit as a reference to evaluate the performance of 4 different *C. trachomatis* antigens in LISA. The results should be interpreted carefully. Second, the *C. trachomatis* NAAT positive sera were all from women, we did not evaluate the influence of gender on assay performance. Nevertheless, our previous longitudinal study on the seroepidemiology of *C. trachomatis* infection in the general population of China revealed no significant gender differences in terms of the prevalence of anti-*C. trachomatis* antibody (72). Third, the anti-*C. pneumoniae* antibody status was determined by only one commercial *C. pneumoniae* ELISA without additional assessment. Fourth, the serum IgG concentration may be different in adults and young children, which may affect the results of the analysis. Therefore, further studies are needed to assess the contribution of gender and *C. pneumoniae* to the performance of *C. trachomatis* LISA. In addition, the Pgp3 protein may highly conserve in plasmids of most other *Chlamydia* species that infect animals (73) and result in cross-reactive in the human sera that exposure to the Chlamydia from animals. However, the incidence of *C. psittaci* pneumonia may be very low. For example, a systematic review and meta-analysis showed that pneumonia caused by *C. psittaci* accounted for only 1% of community-acquired pneumonia cases (74). A multi-center observational study in China showed that, in a total of 4,545 patients with complicated or atypical pulmonary infection, the prevalence of *C. psittaci* was determined to be 2.1% (96/4545) by using metagenomic next generation sequencing, suggesting that prevalence of parrot fever remains low and sporadic in China (75). Finally, anti-Pgp3 may be absent in certain pgp3 plasmid-free *C trachomatis* strains (76, 77), which is a limitation of our manuscript. Fortunately, previous studies indicate that infection with plasmid-free *C. trachomatis* is rare in the general population (78, 79). We should still interpret the findings with caution.

In conclusion, we evaluated four *C. trachomatis* species-specific antigens Pgp3, TmeA, and InaC, and HSP60 in our LISA platform to detect anti-*C. trachomatis* and confirmed the great performance and diagnostic efficiency of Pgp3-based LISA. Our results indicate that Pgp3 can be used in *C. trachomatis* screening in both high-risk subjects and low-risk general population.

## Data availability statement

The raw data supporting the conclusions of this article will be made available by the authors, without undue reservation.

## Ethics statement

The studies involving humans were approved by Ethics Committee of Dermatology Hospital of Southern Medical University. The studies were conducted in accordance with the local legislation and institutional requirements. Written informed consent for participation in this study was provided by the participants’ legal guardians/next of kin.

## Author contributions

YP: Data curation, Formal analysis, Investigation, Methodology, Writing – original draft. JS: Data curation, Formal analysis, Investigation, Methodology, Writing – original draft. CL: Investigation, Resources, Writing – review & editing. YL: Investigation, Resources, Writing – review & editing. HC: Investigation, Resources, Writing – review & editing. ST: Conceptualization, Funding acquisition, Project administration, Writing – review & editing.
