# Supplementary material for: The serodiagnositic value of Chlamydia trachomatis antigens in antibody detection using luciferase immunosorbent assay
Source: Front Public Health. 2024 Feb 27;12:1333559. doi: 10.3389/fpubh.2024.1333559 (PMC10927828; doi:10.3389/fpubh.2024.1333559)

**Supplementary Table S1. Characteristics of study populations**

| Populations | No.  Tested | Gender (n, %) | | Age (year) (Median, range) |
| --- | --- | --- | --- | --- |
|  |  | Female | Male |  |
| Group 1^a^ | | | | |
| Current CT-infected women | 125 | 125 (100) | 0 | 29 (18 - 55) |
| Children with low risk of exposure to CT | 125 | 60 (48) | 65 (52) | 3 (1 - 6) |
| Group 2^b^ | | | | |
| General adults | 200 | 100 (50) | 100 (50) | 25 (18 - 44) |

a: The current *C. trachomatis*-infected women were confirmed by nucleic acid amplification test (NAAT) and were considered *C. trachomatis* exposure positive. *C. trachomatis* negative samples confirmed by commercial anti*-C. trachomatis* IgG ELISA were collected from healthy, low risk children aged 1 to 6 years old and were assumed as *C. trachomatis* un-exposed.

b: The samples were collected from 200 general adults while their *C. trachomatis* exposure status were not known.

**Supplementary Table S2. *C. trachomatis* antigens used in this study**

| Gene | Protein | Amino acid | | Recognition frequency ^a^ (%) | CT D-K genotypes ^b^ | | Species-specific protein |
| --- | --- | --- | --- | --- | --- | --- | --- |
|  |  | Sequence | Length (aa) |  | Sequence ^c^ conservation (%) | Probability  of reactivity ^d^ (%) |  |
| pORF5 | Pgp3, plasmid-encoded protein 3 | MGNSG------^e^PQTNA | 264 | 96 | 98.7 | 94.0 | Yes |
| CT694 | TmeA, translocated membrane-associated effector A | MSIRP------ SVFLD | 323 | 81 | 97.8 | 93.3 | Yes |
| CT813 | InaC, inclusion membrane protein for actin assembly | MTTLP------ RRGSI | 264 | 79 | 98.9 | 94.1 | Yes |
| CT110 | HSP60, heat shock protein 60 | MVAKN------AGMDY | 544 | 47 | 99.9 | 94.8 | No |

a: The recognition frequency of *C. trachomatis* NAAT positive patients was determined by Wang et al. (ref)

Ref: Wang, J., Zhang, Y., Lu, C., Lei, L., Yu, P. and Zhong, G. (2010). A genome-wide profiling of the humoral immune response to Chlamydia trachomatis infection reveals vaccine candidate antigens expressed in humans. *J Immunol* 185(3), 1670-1680. doi: 10.4049/jimmunol.1001240.

b: The reference sequences used here are D/AE001273.1, E/NC_020971.1, F/NZ_CP010569.1, G/NC_020941.1, H/NZ_CP017733.1, I/CP010571.1, J/CP006680.1, K/HE601794.1.

c: Average percentage of amino acid sequence identity within 8 *C. trachomatis* strains representing CT D-K genotypes.

d: Average probability of reactivity with sera against *C. trachomatis* D-K genotypes is calculated by amino acid sequence identity using *P*_cross_ = e ^(-9.4153 +0.123223 x percent sequence identity)^/ (1 + e ^(-9.4153+0.123223 x percent sequence identity)^).

e: The rest amino acid sequences of the *C. trachomatis* antigens used in this study were labelled as “-----”.

**Supplementary Table S3. Performance of anti-*C. trachomatis* antibody assay according to likelihood ratios ^a^**

| Assays | Strong diagnostic efficiency | | Moderate diagnostic efficiency | | Poor diagnostic  efficiency | |
| --- | --- | --- | --- | --- | --- | --- |
|  | Specificity  (%) | Sensitivity  (%) | Specificity  (%) | Sensitivity  (%) | Specificity  (%) | Sensitivity  (%) |
| **Sera from active *C. trachomatis*-infected women and anti-*C. trachomatis* negative children with low risk of exposure to *C. trachomatis*** | | | | | | |
| Mikrogen | 91 - 99 | 97.8 - 94.2 | 85 - 99 | 98.4 - 94.2 | 70 - 99 | 99.1 - 94.2 |
| Pgp3 | 91 - 99 | 96.6 - 92.8 | 85 - 99 | 97.4 - 92.8 | 70 - 99 | 98.4 - 92.8 |
| TmeA | 91 - 96 | 93.9 - 91.1 | 85 - 99 | 95.5 - 85.6 | 70 - 99 | 97.5 - 85.6 |
| InaC | 91 - 98 | 93.9 - 90.2 | 85 - 99 | 95.1 - 88.5 | 70 - 99 | 96.7 - 88.5 |
| HSP60 | 91 - 95 | 93.8 - 90.8 | 85 - 97 | 96.0 - 87.7 | 70 - 99 | 98.3 - 80.2 |
| **Sera from healthy blood donors with unknown anti-*C. trachomatis* status ^b^** | | | | | | |
| Pgp3 | 91 - 99 | 99.7 - 95.2 | 85 - 99 | 99.9 - 95.2 | 70 - 99 | 100.0 - 95.2 |
| TmeA | 91 - 94 | 92.2 - 90.9 | 85 - 99 | 93.7 - 85.3 | 70 - 99 | 95.7 - 85.3 |
| InaC | - | - | 85 - 88 | 88.5 - 87.1 | 70 - 97 | 92.9 - 77.7 |
| HSP60 | - | - | - | - | 70 - 80 | 88.4 - 82.0 |

a: Likelihood ratios were used to define performance of the assays, i.e. strong (+LR ≥ 10, -LR ≤ 0.1), moderate (+LR ≥ 5, -LR ≤ 0.15) and poor (+LR ≥ 2.5, -LR ≤ 0.25) diagnostic efficiency.

b: Compared with Mikrogen ELISA results.

**Supplementary Table S4. The prevalence ranges of anti-*C. trachomatis* IgG assays with different performance according to predictive analysis ^a^.**

| Assays | Specificity (%) | Sensitivity (%) | Antibody prevalence range (%) | | |
| --- | --- | --- | --- | --- | --- |
|  |  |  | High performance | Moderate  performance | Poor performance |
| **Sera from active *C. trachomatis*-infected women and anti-*C. trachomatis* negative children with low risk of exposure to *C. trachomatis*** | | | | | |
| Mikrogen |  |  |  |  |  |
|  | 99 | 94.2 | 10-63 | 6-77 | 3-87 |
|  | 98 | 95.5 | 18-68 | 10-81 | 5-89 |
|  | 95 | 97.0 | 34-75 | 21-86 | 12-92 |
|  | 90 | 97.9 | 51-81 | 34-89 | 21-94 |
| Pgp3 |  |  |  |  |  |
|  | 99 | 92.8 | 10-57 | 6-73 | 3-84 |
|  | 98 | 94.1 | 18-62 | 10-76 | 6-86 |
|  | 95 | 95.7 | 35-68 | 21-81 | 12-89 |
|  | 90 | 96.8 | 51-73 | 35-84 | 21-91 |
| TmeA |  |  |  |  |  |
|  | 99 | 85.6 | 11-40 | 6-58 | 3-73 |
|  | 98 | 88.5 | 19-45 | 11-62 | 6-77 |
|  | 95 | 91.9 | 36-54 | 22-70 | 12-82 |
|  | 90 | 94.3 | 52-61 | 35-75 | 21-86 |
| InaC |  |  |  |  |  |
|  | 99 | 88.5 | 11-46 | 6-63 | 3-77 |
|  | 98 | 90.2 | 19-50 | 10-66 | 6-80 |
|  | 95 | 92.5 | 36-55 | 22-71 | 12-83 |
|  | 90 | 94.2 | 52-60 | 35-75 | 21-86 |
| HSP60 |  |  |  |  |  |
|  | 99 | 80.2 | 12-33 | 6-50 | 4-66 |
|  | 98 | 85.1 | 20-39 | 11-56 | 6-72 |
|  | 95 | 90.8 | 36-50 | 22-67 | 13-80 |
|  | 90 | 94.3 | 52-61 | 35-76 | 21-86 |
| **Sera from healthy blood donors with unknown anti-*C. trachomatis* status ^b^** | | | | | |
| Pgp3 |  |  |  |  |  |
|  | 99 | 95.2 | 10-67 | 5-80 | 3-89 |
|  | 98 | 97.5 | 18-79 | 10-88 | 5-93 |
|  | 95 | 99.2 | 34-92 | 21-95 | 12-97 |
|  | 90 | 99.8 | 51-97 | 34-98 | 21-99 |
| TmeA |  |  |  |  |  |
|  | 99 | 85.1 | 11-40 | 6-57 | 3-72 |
|  | 98 | 87.5 | 19-43 | 11-61 | 6-75 |
|  | 95 | 90.4 | 36-49 | 22-66 | 13-79 |
|  | 90 | 92.5 | 52-54 | 36-70 | 22-82 |
| InaC |  |  |  |  |  |
|  | 99 | 70.2 | 13-24 | 7-40 | 4-57 |
|  | 98 | 75.0 | 22-28 | 12-43 | 7-61 |
|  | 95 | 81.2 | - ^c^ | 24-50 | 14-66 |
|  | 90 | 85.9 | - | 37-56 | 23-71 |
| HSP60 |  |  |  |  |  |
|  | 99 | 34.7 | - | 13-23 | 7-37 |
|  | 98 | 43.9 | - | 19-25 | 11-41 |
|  | 95 | 58.2 | - | 31 | 18-47 |
|  | 90 | 70.1 | - | - | 27-54 |

a: High performance, both positive predictive value (PPV) and negative predictive value (NPV) ≥ 90.9% ; moderate performance, PPV and NPV ≥ 83.3%; poor performance, PPV and NPV ≥ 71.4%.

b: Compared with Mikrogen ELISA results.

c: -, indicating that no antibody prevalence for the assay at selected specificity can achieve the acquired performance.

**Figure Legends**

**Supplementary Figure S1.** **Anti-*C. trachomatis* IgG determined by Mikrogen ELISA and *C. trachomatis* antigen-based LISAs for sera from active *C. trachomatis*-infected women and the children with low risk of exposure to *C. trachomatis*.** Sera from 125 women with active *C. trachomatis* infection (represented by solid circle) and 125 *C. trachomatis* negative children (solid triangle) were tested by commercial Mikrogen ELISA and *C. trachomatis* antigen-based LISAs. The observed value of *C. trachomatis*-antigen LISAs was shown as relative luciferase unit (RLU) while Mikrogen was shown as units. Red lines indicated the cut-off values of individual assays. The cutoff value were 4.1 Log_2_RLU for Pgp3-LISA, 0.7 Log_2_RLU for TmeA-LISA, 1.7 Log_2_RLU for InaC-LISA, 1.6 Log_2_RLU for HSP60-LISA, and 24 units for Mikrogen. Green solid circles indicate active *C. trachomatis*-infected women who was anti-*C. trachomatis* antibody negative. Specifically, there were 9 sera from active *C. trachomatis*-infected women were negative for Pgp3-LISA, 14 for TmeA-LISA, 12 for InaC-LISA, and 7 for HSP60-LISA, and 8 for Mikrogen ELISA. The 7 sera from active infected women were those that were negative by both the ELISA and the four LISA methods. Red solid triangles indicate the children with low risk of exposure to *C. trachomatis* who was anti-*C. trachomatis* antibody positive.


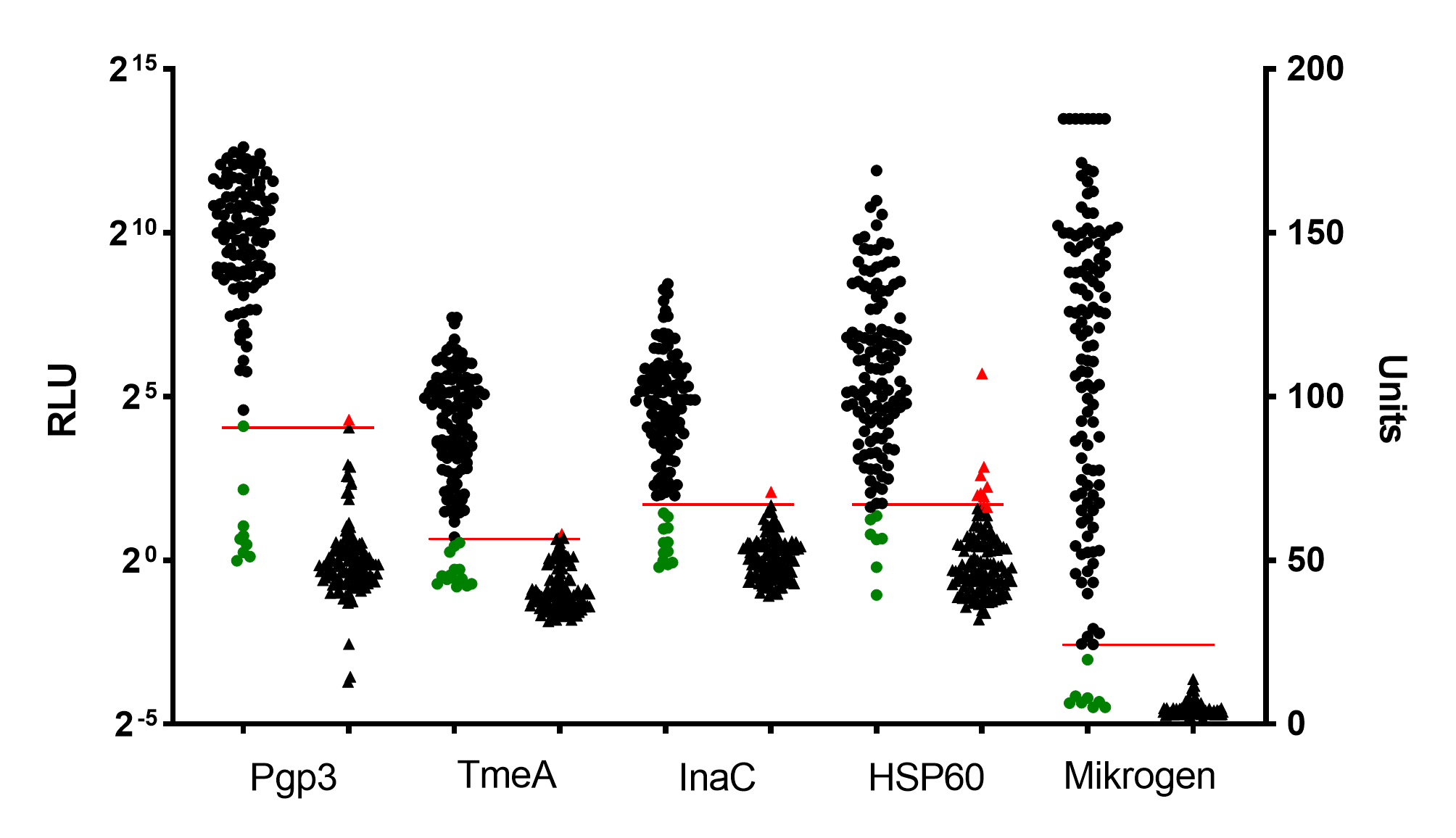

Supplement: Supplementary file 1 [file Data_Sheet_1.docx]
